# Implementation and evaluation of infection prevention and control training in sub-Saharan Africa

**DOI:** 10.1016/j.infpip.2025.100485

**Published:** 2025-10-04

**Authors:** D. Odada, J. Ndai, R. Thuku, R. Adam

**Affiliations:** a Department of Nursing, Aga Khan University Hospital, Nairobi, Kenya; b Department of Quality, Aga Khan University Hospital, Nairobi, Kenya; c Department of Internal Medicine, Aga Khan University Hospital, Nairobi, Kenya; d Department of Pathology, Aga Khan University Hospital, Nairobi, Kenya

**Keywords:** Training, Evaluation, Learning outcome, Infection preventionists

## Abstract

Healthcare-associated infections remain a global concern, exacerbated by limited competent infection prevention and control personnel in low- and middle-income countries (LMICs). This study evaluated infection prevention and control training using Kirkpatrick's model to determine its effectiveness in enhancing competence in infection preventionists in Kenya. This descriptive design assessed participants' knowledge and satisfaction with a training workshop through pre- and post-tests and a five-point Likert scale. Forty participants showed significant improvement in knowledge (pre-test: 49%, post-test: 64%; *P*<0.05) and high satisfaction with the training workshop (mean 4.68/5). The findings affirm the effectiveness of structured training in LMICs to enhance competency, and underscore the need for formal professional development.

## Introduction

Infection prevention and control (IPC) is a cornerstone in health care globally. Competency in implementing IPC programmes is imperative [1]. Competent IPC professionals are required to achieve effective IPC programmes [2]. In high-income countries, IPC professionals have clearly defined career pathways, supported through access to structured training and professional certification [3]. In comparison, in most African countries, professional pathways for healthcare staff interested in IPC are undefined and opportunities are limited [4]. In the USA, a survey found that only 50% of hospital-based infection preventionists (IPs) had access to organization-based training and mentorship, and the situation is thought to be even more critical in low- and middle-income countries (LMICs) where support systems are often non-existent [5,6]. Additionally, IPC fields are often not recognized as a formal specialty, limiting training to only a few countries that offer it as an academic programme [7]. As a result, most healthcare facilities have failed to meet the minimum competency standards for IPs set by the World Health Organization [1].

Given the need to design and implement tools to develop IP competence, Aga Khan University Hospital Nairobi (AKUHN) has been conducting annual training sessions in IPC, targeting healthcare professionals actively involved in IPC programmes [6]. The content of these sessions is based upon international best practices (with special consideration of regional epidemiology and IPC practices) [2]. AKUHN is accredited by Joint Commission International, meeting international standards for IPC programmes and practice. The primary aim of this study was to evaluate the effectiveness of the IPC training workshop using Levels 1 and 2 of the Kirkpatrick model to assess participant satisfaction and learning outcomes.

## Methods

This descriptive study evaluated the outcomes of a 2-week IPC workshop in June 2024. The study applied Levels 1 and 2 of the Kirkpatrick model, focusing on participants' feedback on the workshop and their learning outcomes [8]. In total, 40 participants were eligible for training by virtue of being involved in an IPC programme. (Figure S2, see supplementary on eligibility)

The workshop was organized by a team that included one infectious diseases physician and three Level-3-certified IPs, alongside 15 other professionals facilitating the training. Instruction was delivered through a blend of didactic lectures (80%) and practical sessions (20%), incorporating simulation-based learning in a university laboratory and visits to hospital specialty services areas. Participants were recruited through their facility leadership, targeting healthcare workers involved in IPC programmes. A registration fee of $120 was applied and covered by the participants' respective institutions. A structured feedback form consisting of 10 Likert-scale questions (ranging from ‘strongly agree’ to ‘strongly disagree’) was used to assess participant satisfaction with the workshop. Knowledge retention was assessed using pre- and post-tests, each consisting of 50 randomly selected multiple-choice questions covering the eight core IPC competency domains. Each test was administered over a duration of 1 h. (See supplementary material for additional details on methods)

Statistical analyses were performed using SPSS Version 24 (IBM Corp., Armonk, NY, USA). Descriptive statistics are presented as mean and 95% confidence interval (CI) for continuous variables, and median and interquartile range (IQR) for ordinal data. Pre- and post-test scores were compared using paired sample *t*-tests. Significance was determined using a two-tailed *P*-value threshold of <0.05. Ethical approval was granted by AKUHN Institutional Scientific and Ethics Review Committee [2024/ISERC-61 (v3)] and licensed by the National Commission of Science, Technology and Innovation, (NACOSTI/P/24/36787). All participants provided written informed consent.

## Results

The IPC training workshop included 40 participants with diverse demographic characteristics. The median age group of the participants was 38–47 years (IQR 28–47 years), and 80% were female. Thirteen percent of participants had a Master's degree, 50% had a Bachelor's degree, and 38% had diploma-level training. Most (90%) of the participants had a nursing background. Half of the participants were full-time IPC staff, and the remainder (non-full-time IPC staff) were members of IPC committees and IPC link staff. Among the full-time IPC staff, 70% had 3–5 years of experience and 30% had 1–3 years of experience in their roles. While participants were drawn from various facilities, the majority (43%) were from tertiary private facilities, while 28% were from tertiary public facilities. Most participants (65%) worked in facilities with a bed capacity of 250–500 beds.

### Participant satisfaction

Participant satisfaction with the training workshop was evaluated using a five-point Likert scale, with 10 key statements assessing various aspects of the training experience. Feedback items included the extent to which training objectives were met, trainer engagement, topic relevance, content organization, trainer preparedness, training length, pacing, interaction, venue suitability, and the usefulness of the training for participants' work. The highest-rated aspects were trainer engagement, topic relevance, and participant interaction; each with a mean score of 4.88 (95% CI 4.75–4.98). The lowest-rated item was training length, which had a mean score of 4.13 (95% CI 3.80–4.40), with some participants suggesting the need to increase the length of training. (Table S1, see supplementary material)

### Learning outcomes

The mean pre- and post-test scores were 49% and 64%, respectively (*P*<0.05, see Table I). Relative to their baseline scores, most participants (95%) recorded improved scores on the post-test (Figure S1, see online supplementary material). The Certification Board of Infection Control and Epidemiology domains assessed are outlined in Table I. The differences between pre- and post-test scores for four categories – surveillance and epidemiological investigation, employee/occupational health, management and communication, and education and research – were significant (*P*<0.05). Participants demonstrated improved post-test scores across all groups (Table II). However, there were no significant differences in score improvements between full-time IPC staff and non-full-time IPC staff. Similarly, participants with >3 years of IPC experience did not significantly outperform those with fewer years of IPC experience.

**Table I:** Mean scores for each category

Categories	Proportionof questions	Pre-test Mean (95% CI) *N*=40	Post-test Mean (95% CI) *N*=40	*P*-value(two-tailed)
All	100%	49% (46–51%)	64% (60–68%)	<0.005
Infectious disease processes	18%	59% (53–64%)	66% (59–73%)	0.104
Surveillance and epidemiological investigation	18%	53% (48–58%)	68% (62–74%)	0.000
Preventing/controlling transmission of infectious agents	18%	52% (46–58%)	57% (50–64%)	0.256
Employee occupational health	7%	56% (49–63%)	71% (61–82%)	0.025
Management and communication	11%	5% (3–8%)	59% (52–67%)	<0.005
Education and research	7%	32% (24–40%)	72% (66–78%)	<0.005
Environment of care	8%	49% (41–58%)	55% (47–62%)	0.262
Cleaning disinfection, and sterilization ofmedical devices and equipment	13%	47% (40–55%)	55% (49–61%)	0.134

CI, confidence interval.

**Table II:** Pre- and post-test scores for infection prevention and control (IPC) staff and years in practice

	*N*	Pre-test Mean (95% CI)	Post-test Mean (95% CI)	*P*-value (two-tailed)
Full-time IPC staff	20	49.20 (44.67–54.00)	64.40 (58.57–70.72)	<0.0001
Non-full-time IPC staff^a^	20	48.3 (45.47–50.71)	63.10 (57.18–69.78)	0.0005
0–3 years of practice	6	47.80 (43.77–52.52)	62.30 (57.65–67.52)	<0.0001
>3 years of practice	14	49.70 (46.10–53.36)	65.20 (58.21–72.87)	0.0004

CI, confidence interval.

a Members of IPC committees and IPC link staff.

## Discussion

The IPC training under study was demonstrated to be satisfactory in meeting all aspects of the feedback items. This is consistent with results from a systematic review, which showed that student participants across all studies reacted positively towards their training experience [9]. Non-significant knowledge gain in some topics may reflect potential limitations in IPC budget allocation, sterilization infrastructure, and lack of involvement in construction and renovation assessments. The positive impact of the training workshop on participants' knowledge in all IPC competencies represents a similar finding to a study in Pakistan, which also evaluated the satisfaction of trainees with IPC training [8]. The observed difference indicated a notable increase in knowledge from baseline, consistent with findings in England and Guinea that evaluated training in IPC and demonstrated substantial improvement in knowledge among participants in the post-test [10]. However, some of the present findings contrasted with the study in Pakistan, in which IPC trainers and supervisors had a higher baseline level of knowledge compared with other healthcare professionals. In the present study, there was no difference in baseline knowledge between full-time IPC staff and non-full-time IPC staff [10]. This discrepancy could be due to differences in individual facilities, IPC training structures, and resources. Another reason could be the lack of minimum criteria for appointing IPs, and limited training, mentorship and continuing professional development for IPs in their roles after being hired. The relevance of the training content was rated highly, indicating strong alignment with professional development needs. This suggested that participants' training expectations were met, concurring with a qualitative finding which highlighted the significance of IPC career path development, particularly in low-resource settings, where structured professional growth opportunities may be limited [6]. This underscores the need for sustained investment in IPC education to enhance knowledge of IPs and contribute to advancement of their careers. Similar to a previous study, the present findings demonstrated significant overall improvement in participants' knowledge. This supports the effectiveness of targeted training which aligns with structured learning, and the value of such training in equipping novice IPs to meet core IPC competencies [5]. Despite an overall gain in knowledge, the mean scores did not meet the threshold comparable with credentialling bodies. The effectiveness of the training workshop may have been influenced by content complexity and relevance to participants' work context, suggesting the adoption of differentiated teaching approaches, foundational reinforcement for strong-baseline topics, and intensive capacity-building for weaker areas in future training.

In conclusion, training IPs in best practices, particularly in resource-limited settings, contributes significantly to building a competent and flexible IPC workforce. This study provides a roadmap for evaluating IP training, and identifies opportunities for IPs from diverse backgrounds and settings to enhance their competencies in the dynamic field of IPC. The findings support the need to include a broader participant base in future training workshops, formalize training, and provide ongoing professional development and supportive structures to build competency in IPs. The training was limited in terms of assessing the practical application of skills by participants. Future training should explore methods that track participants' ability to apply knowledge in implementing infection prevention programmes.

## Funding sources

None.

## Conflicts of interest

None declared.

## References

[1] Storr J., Twyman A., Zingg W., Damani N., Kilpatrick C., Reilly J. (2017). Core components for effective infection prevention and control programmes: new WHO evidence-based recommendations. Antimicrob Resist Infect Control.

[2] Bubb T.N., Billings C., Berriel-Cass D., Bridges W., Caffery L., Cox J. (2016). APIC professional and practice standards. Am J Infect Control.

[3] Loveday H. (2017). Credentialing, competence and certification: infection prevention and control as a specialty. J Infect Prevent.

[4] Kamara I.F., Tengbe S.M., Fofanah B.D., Bunn J.E., Njuguna C.K., Kallon C. (2022). Infection prevention and control in three tertiary healthcare facilities in Freetown, Sierra Leone during the COVID-19 pandemic: more needs to be done!. Int J Environ Res Public Health.

[5] Holmes K., Boston K.M., McCarty J., Steinfeld S., Kennedy V. (2024). Enhancing infection preventionist certification success through a structured training program. Am J Infect Control.

[6] Tomczyk S., Storr J., Kilpatrick C., Allegranzi B. (2021). Infection prevention and control (IPC) implementation in low-resource settings: a qualitative analysis. Antimicrob Resist Infect Control.

[7] Mehtar S., Wanyoro A., Ogunsola F., Ameh E.A., Nthumba P., Kilpatrick C. (2020). Implementation of surgical site infection surveillance in low-and middle-income countries: a position statement for the International Society for Infectious Diseases. Int J Infect Dis.

[8] Savul S., Ikram A., Khan M.A., Khan M.A. (2021). Evaluation of infection prevention and control training workshops using Kirkpatrick's model. Int J Infect Dis.

[9] Aryadoust V. (2017). Adapting Levels 1 and 2 of Kirkpatrick's model of training evaluation to examine the effectiveness of a tertiary-level writing course. Pedagogies.

[10] Soeters H.M., Koivogui L., de Beer L., Johnson C.Y., Diaby D., Ouedraogo A. (2018). Infection prevention and control training and capacity building during the Ebola epidemic in Guinea. PLoS One.

