# Supplementary material for: Implementation and evaluation of infection prevention and control training in sub-Saharan Africa
Source: Infect Prev Pract. 2025 Oct 4;7(4):100485. doi: 10.1016/j.infpip.2025.100485 (PMC12595373; doi:10.1016/j.infpip.2025.100485)
Supplement: Multimedia component 1 [file mmc1.docx]

**Supplementary material**

**Methods**

The training content was developed using the APIC Competency Model and aligned with both local and international IPC guidelines, including those from the CDC, WHO, and the APIC Text. Questions for the pre- and post-tests were drawn from the APIC Student Guide question bank, covering eight core IPC competency domains. A total of 50 questions were randomly selected using the RAND function in Microsoft Excel. Planning for the training began three months in advance, with biweekly meetings held by the organizing team. The workshop was facilitated by a multidisciplinary team of experts, including infectious disease physicians, microbiologists, occupational health specialists, quality and risk management professionals, and informatics and data management experts.

**Table S1**

**Participant Reaction**

| Reaction Item | Mean (95% CI)  N=40 |
| --- | --- |
| The objectives of the training were met. | 4.68 (4.43-4.88) |
| The trainers were engaging. | 4.88 (4.75-4.98) |
| The topics covered were relevant. | 4.88 (4.75-4.98) |
| The content was organized and easy to follow. | 4.78 (4.65-4.9) |
| The trainers were well prepared and able to answer any questions. | 4.73 (4.58-4.85) |
| The training length was appropriate. | 4.13 (3.8-4.4) |
| The pace of the training was appropriate for the content and attendees. | 4.58 (4.35-4.77) |
| Participation and interaction were encouraged. | 4.88 (4.75-4.98) |
| The venue was appropriate for the event. | 4.65 (4.47-4.82) |
| The training experience will be useful in my work. | 4.85 (4.73-4.95) |

Five-point Likert scale ranging from strongly disagree, disagree, neutral, agree, strongly agree, and not applicable

**Figure S1. Individual scores for pre- and post-tests**

**Figure S2:** **IPC Training workshop.**
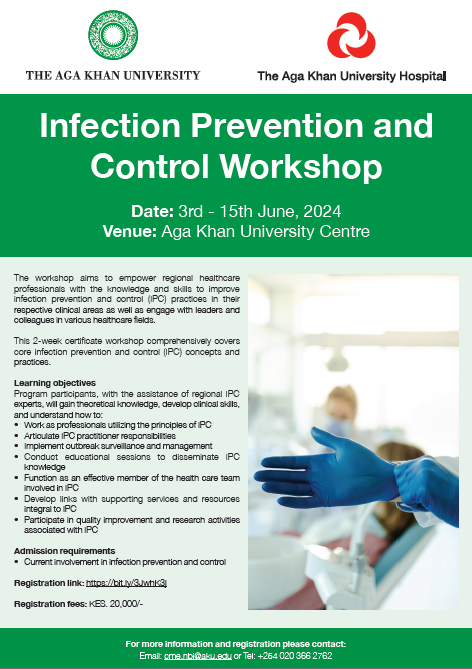


**IPC WORKSHOP TOPICS**

Identification of Infectious Disease Processes

- Basic Microbiology
- Pathogens and Diseases: Measles, Cholera, CRE Klebsiella, TB, Respiratory viruses
- Blood Culture: A Key Investigation for Diagnosis of Bloodstream Infections
- Hospital Antimicrobial Stewardship Programs: The Core Elements
- Emergency Preparedness: Communicable Diseases
- Surge Preparedness: Respiratory Illnesses
- Function of Simulation and drills for IPC

Surveillance and Epidemiologic Investigation

- Epidemiology and Surveillance
- NHSN Definitions for HAIs
- Outbreak Management
- External and Internal Benchmarking for HAI surveillance
- Monitoring Hand Hygiene Compliance
- Communicable Disease Screening
- Process and Outcome monitoring for IPC programs.
- Surgical Site infection Surveillance: Organizing a Program to Improve Surgical Site Outcomes
- Monitoring and Prevention of Intravascular Catheter-Related Infections
- Monitoring and Prevention of Catheter-Associated Urinary Tract Infections
- Monitoring and Prevention of Healthcare-Associated Respiratory Tract infections

Preventing/Controlling the Transmission of Infectious Agents

- Developing Risk Registers for IPC Programs
- Introduction to Standard Precautions
- Hand Hygiene: Why, How and When?
- Transmission-based Precautions
- Infection Control Aspects in Laundry
- Infection Control Aspects in Food and Nutrition
- IPC in Specialty Units: Dialysis, Oncology, NICU, Critical care

Employee/Occupational Health

- Infection Prevention and Control Program: What is your role?
- Infection Prevention and Control aspects of Occupational Health

Management and Communication

- What facilitates a Successful Infection Prevention and Control program.
- WHO Guidelines on Core Components of Infection Prevention and Control Programs
- Conducting a Facility Assessment for the Infection Prevention Control Program: IPCAF Guide by WHO
- Infection prevention and Control related sentinel events
- Monitoring IPC programs: RCA, PDSA, SWOT Analysis
- Quality performance improvement and patient safety

Education and Research

- Data management to support Infection Prevention and Control
- Informatics support in Infection Prevention and Control
- Principles of Auditing
- Audit tools for IPC programs.
- Basics of Research
- Applying teaching and learning concepts in IPC

Environment of Care

- Infection Control Risk Assessment in Construction and Renovation in Health Facilities: A strategy in Prevention of Construction-Related Healthcare Infections
- Environmental Cleaning & Disinfection and Waste Management

Cleaning, Disinfection, and Sterilization of Medical Devices and Equipment

- Environmental Cleaning & Disinfection and Waste Management
- Decontamination and Reprocessing of Medical Devices

Hospital Units Tours for had on experience

- Tours: Laundry, Kitchen, CSSD
- Tours: Dialysis, Critical care unit, oncology, microbiology unit Evaluation
- Assessing and evaluation Products for Infection Prevention
- Simulation Lab
